# Prediction of Failure Pressure of Sulfur-Corrosion-Defective Pipelines Based on GABP Neural Networks

**DOI:** 10.3390/ma18133177

**Published:** 2025-07-04

**Authors:** Li Zhu, Yi Xia, Bin Jia, Jingyang Ma

**Affiliations:** 1 School of Civil Engineering and Architecture, Southwest University of Science and Technology, Mianyang 621010, China; zhuli@swust.edu.cn (L.Z.); jiabin216@126.com (B.J.); majy0617@icloud.com (J.M.); 2 China Electronic System Engineering Co., Ltd., Beijing 100071, China

**Keywords:** sulfur-containing corrosion, defective pipelines, neural networks, failure prediction

## Abstract

This study systematically investigates the degradation and failure prediction of pipeline materials in sulfur-containing environments, with a particular focus on X52 pipeline steel exposed to high-sulfur environments. Through uniaxial tensile tests to assess mechanical properties, it was found that despite surface corrosion and a reduction in overall structural load-bearing capacity, the intrinsic mechanical properties of X52 steel did not exhibit significant degradation and remained within standard ranges. The Johnson–Cook constitutive model was developed to accurately capture the material’s plastic behavior. Subsequently, a genetic algorithm-optimized backpropagation (GABP) neural network was employed to predict the failure pressure of defective pipelines and the corrosion rate in acidic environments, with prediction errors controlled within 5%. By integrating the GABP model with NACE standard methods, a framework for predicting the remaining service life for in-service pipelines operating in sour environments was established. This method provides a novel and reliable approach for pipeline integrity assessment, demonstrating significantly higher accuracy than traditional empirical models and finite element analysis.

## 1. Introduction

With the ongoing advancement of global oil and gas exploration into deeper and more acidic reservoirs, sour natural gas has emerged as a significant unconventional resource [1]. However, corrosion issues arising during its extraction and transportation have become increasingly severe. In particular, under the combined effects of H_2_S and CO_2_, metallic pipelines are highly susceptible to electrochemical corrosion, leading to wall thinning, defect propagation, and a consequent reduction in internal pressure-bearing capacity. In extreme cases, this may result in perforation, leakage, or even catastrophic explosions [2]. Due to the specific environmental conditions and transported media, sour gathering and transportation pipelines are highly susceptible to various forms of degradation, including material corrosion, hydrogen-induced cracking (HIC), and sulfide stress cracking (SSC). The primary form of corrosion arises from electrochemical reactions involving sulfides and carbon dioxide dissolved in water. This leads to widespread metal thinning as well as the formation of corrosion pits and groove-like defects. These corrosion defects result in local stress concentrations under internal pressure, significantly reducing the pipeline’s load-bearing capacity.

HIC and SSC represent more insidious degradation mechanisms, as they do not produce apparent macroscopic defects. Their underlying mechanism involves the dissolution of molecular H_2_S in water, which generates a large amount of atomic hydrogen. This atomic hydrogen diffuses into the metallic microstructure and accumulates at stress concentrators such as crack tips or voids, where it recombines to form molecular hydrogen. The accumulation of hydrogen molecules generates high internal pressures, initiating cracks or hydrogen blisters [3,4,5,6]. Moreover, the presence of hydrogen reduces the cohesive forces between metal atoms, thereby embrittling the material. When the applied tensile stress exceeds a critical threshold, the structure may undergo sudden brittle fracture.

While macroscopic corrosion defects inevitably compromise structural integrity, the intrinsic material strength of the pipeline plays a critical role in integrity assessments under sour service conditions. Therefore, it is essential to investigate the evolution of the material properties of pipelines after long-term exposure to sour environments in order to ensure accurate and reliable evaluations of their remaining structural performance [7,8,9].

At present, failure pressure prediction methods for corroded pipelines primarily fall into two categories: empirical engineering formulas and numerical simulations. Among them, standard assessment models such as DNV RP-F101 and ASME B31G provide practical guidance but are often limited by conservative estimations and narrow applicability [10,11,12]. Finite element analysis (FEA), as a high-precision method, requires extensive parameter input and computational resources, posing challenges in terms of real-time application and cost-effectiveness in engineering practice [11,13]. With the advancement of artificial intelligence, neural networks have demonstrated significant potential in pipeline failure prediction, leakage identification, and design optimization due to their excellent nonlinear mapping capabilities [14,15,16,17]. Compared to traditional assessment standards and empirical formulas, BP neural networks offer notable advantages in pipeline failure prediction due to their strong nonlinear mapping ability and adaptability. While conventional methods often rely on simplified assumptions and limited parameters, BP networks can integrate multiple complex factors and learn directly from data, enabling more accurate and flexible predictions. Additionally, when optimized with algorithms such as genetic algorithms, BP networks can overcome local minima issues and significantly improve predictive performance, making them more suitable for evaluating pipeline integrity under varied and complex conditions [18,19].

To improve the accuracy and reliability of pipeline integrity assessments, a comprehensive investigation was conducted in this study. First, tensile tests were carried out to examine the material degradation of pipeline steel after seven years of service in a high-sulfur environment. Based on the experimental results, a Johnson–Cook constitutive model was developed to characterize the mechanical behavior of the degraded material, providing a foundation for subsequent numerical simulations. Second, the backpropagation (BP) neural network was optimized using a genetic algorithm (GA), resulting in the development of a hybrid GABP (GABP) model with enhanced prediction capability. Finally, by integrating the optimized neural network with the remaining life evaluation methodology proposed in the NACE standard, an intelligent evaluation framework was established to assess the safety of in-service pipelines operating in sour environments.

## 2. The Effect of Sulfur-Containing Corrosion on the Performance of Pipeline Materials

### 2.1. Materials and Methods

To investigate the effect of sulfur-containing corrosion on material properties, mechanical tests were conducted on pipeline materials that had been in service in a sour environment. The subject of this study was a steel pipeline made of X52 material, which had been operational for seven years in a major sour gas field. The extracted natural gas from this field contained hydrogen sulfide (H_2_S), with a volume fraction ranging from 14.6% to 15.66%, and carbon dioxide (CO_2_), with a volume fraction between 8.57% and 14.1%. The operating temperature of the gas was 50 °C, with a flow velocity of 4 m/s, and an internal pressure ranging from 5.2 to 8.2 MPa.

**Figure 1:**
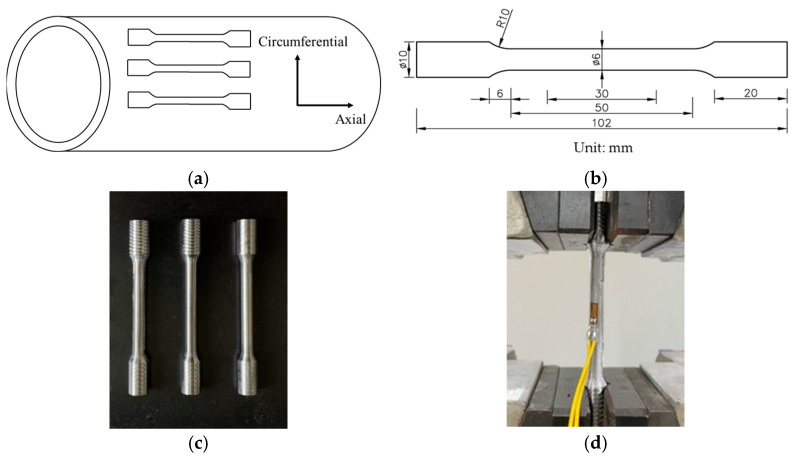
Uniaxial tensile test: (**a**) schematic diagram of specimen sampling; (**b**) geometry of the specimen; (**c**) final machined specimen; (**d**) tensile test setup.

Samples were extracted from the pipeline and subjected to tensile testing to determine the material’s mechanical strength. The dimensions of the tensile specimens are shown in Figure 1a,b. During specimen preparation, electrical discharge machining was first employed to cut raw material billets from the pipeline. Subsequent machining was then performed to remove the 2 millimeter-thick corroded layer from the outer surface, preserving the central portion along the pipe thickness to eliminate the influence of corrosion products and surface morphology on the test results. Throughout the machining process, circulating water was used to cool the specimens, thereby minimizing the thermal effects on the mechanical properties. Ultimately, cylindrical tensile specimens were obtained.

To ensure reliability and comparability, three pipelines of the same material and service conditions but with different diameters and wall thicknesses were tested. The dimensions of these pipelines are listed in Table 1. For each pipeline, three standard tensile specimens were fabricated and tested. The final geometry of the finished specimens is shown in Figure 1c. The tensile tests were conducted as illustrated in Figure 1d using a CMT5150 universal testing machine with a maximum load capacity of 100 kN, and the equipment was sourced from Shenzhen Wance Testing Machine Co., Ltd., Shenzhen, China. The extensometer had a gauge length of 50 mm and a precision class of grade 1. All tests were performed under ambient conditions, with a loading rate of 0.5 mm/min. Strain gauges were attached to the specimens to record strain data, facilitating the accurate determination of the elastic modulus.

### 2.2. Test Results and Analysis

During tensile testing, the cross-sectional area of the specimen continuously changes under load. If conventional engineering stress–strain calculations are used—where engineering stress is defined as the applied load divided by the initial cross-sectional area, and engineering strain is defined as the elongation divided by the initial gauge length—the resulting stress values may be underestimated, which can compromise the accuracy of subsequent modeling. Therefore, to more accurately characterize the material’s mechanical behavior, true stress and true strain should be employed [20]. The corresponding calculation formulas are provided in Equations (1) and (2) below:

(1) $$\sigma_{T\mathrm{ure}}=\frac{FL}{A_{0}L_{0}}$$

(2) $$\varepsilon_{T\mathrm{ure}}=\ln\left(\frac{L}{L_{0}}\right)$$

where *F* is the applied load at the current time; *L* is the specimen length at the current time; *L*_0_ is the initial gauge length of the specimen; and *A*_0_ is the initial cross-sectional area of the specimen.

**Figure 2:**
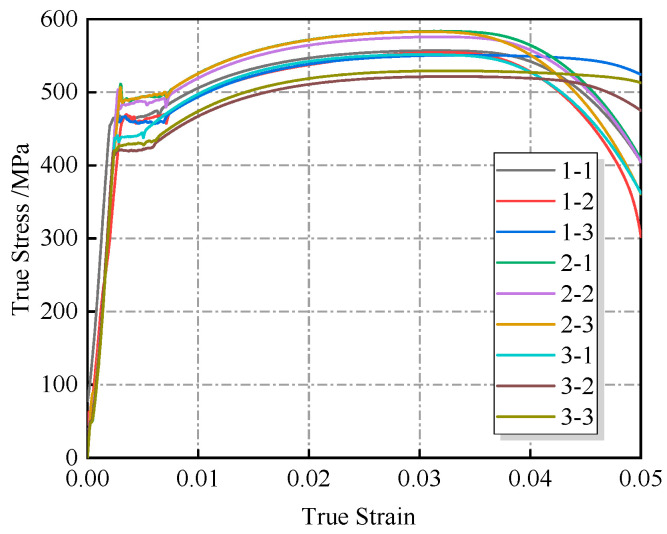
Stress–strain curve of tensile test specimen.

The true stress–strain curves of X52 pipeline steel were obtained by processing the displacement and load data, as shown in Figure 2. The yield strength, tensile strength, and elastic modulus of each specimen are listed in Table 2. A noncorroded control group was not included in this study, as the mechanical properties of X52 steel are already specified in the API pipeline steel standard, which defines a wide range of values: a yield strength between 360 and 530 MPa and a tensile strength between 460 and 755 MPa. Due to the broadness of this range, it was impractical to establish a control group based on other batches of the same grade. However, it can be confirmed that the strength values obtained from the three corroded pipelines tested in this study fall within the standard limits.

According to the existing literature [21], the yield strength and tensile strength of unused X52 pipeline steel from the same gas field are 476.6 MPa and 555.4 MPa, respectively. Compared to the results obtained in this study, the maximum deviation is approximately 10%.

Comprehensive analysis showed that there was no significant degradation in intrinsic mechanical properties. Although corrosion causes a decrease in geometric stability, the test results still meet standard requirements. This indicates that the impact of sulfur corrosion on the load-bearing capacity of X52 pipeline steel is mainly manifested as a decrease in geometric stability rather than a degradation of the material’s inherent properties.

### 2.3. Constitutive Model

The plastic behavior of X52 pipeline steel is described using the conventional J2 flow theory, and the corresponding flow rule is expressed as follows [22]:

(3) $$f\left(\sigma ,\bar{\varepsilon_{p}}\right)=\bar{\sigma }-\bar{\sigma }\left(\bar{\varepsilon_{p}}\right)=0$$

The Johnson–Cook constitutive model was employed to characterize the plastic hardening behavior of X52 steel under quasi-static conditions. This model is widely used due to its computational efficiency and the ease of parameter determination. The constitutive equation is given as follows [23]:

(4) $$\bar{\sigma }=A+B{\left({\bar{\varepsilon }}_{p}\right)}^{n}$$

The stress–strain data prior to necking were selected for analysis. The elastic strain was calculated by dividing the yield strength by the elastic modulus, and the plastic strain was then obtained by subtracting the elastic strain from the total strain. Based on this, the strain hardening curve during the strengthening stage was established. An initial fitting of the Johnson–Cook model parameters A, B, and n was performed using the pre-necking hardening curve. However, these preliminarily fitted parameters could only provide a rough description of the plastic hardening behavior of X52 steel and were associated with significant errors.

**Figure 3:**
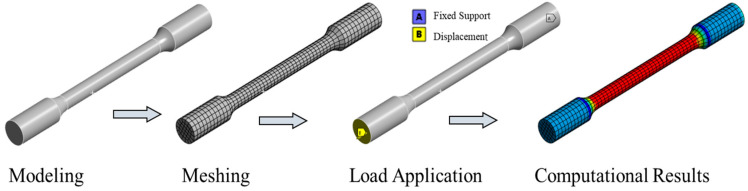
Finite element model of tensile specimen.

Therefore, the initially fitted parameters were imported into a finite element (FE) software to simulate the tensile test. The simulated stress–strain data were extracted and compared with the experimental results. If a significant discrepancy existed between the two curves, so the constitutive parameters were iteratively adjusted. This process was repeated until the maximum agreement between the experimental and simulated curves was achieved. The finite element model is shown in Figure 3.

**Figure 4:**
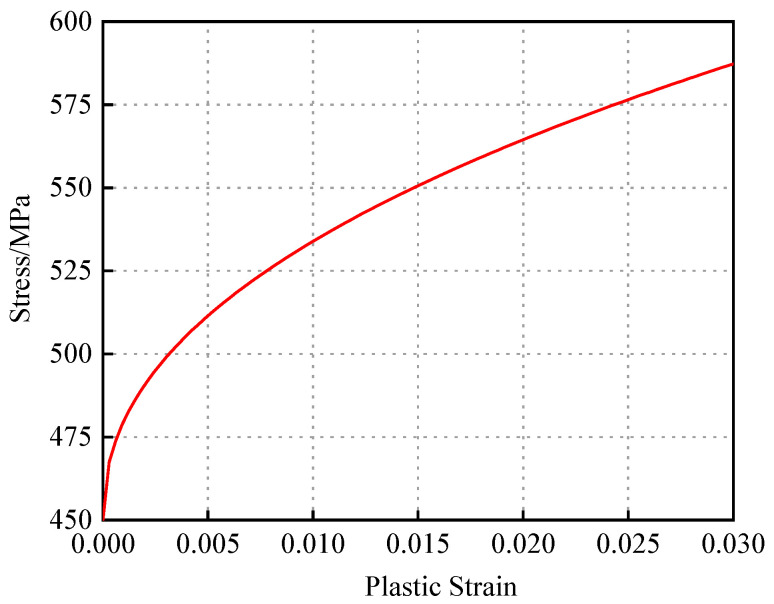
X52 hardening curve.

After multiple iterations of computation and parameter adjustment, the final Johnson–Cook constitutive model for X52 pipeline steel was obtained, as expressed in Equation (5). The corresponding strain hardening curve is illustrated in Figure 4.

**Figure 5:**
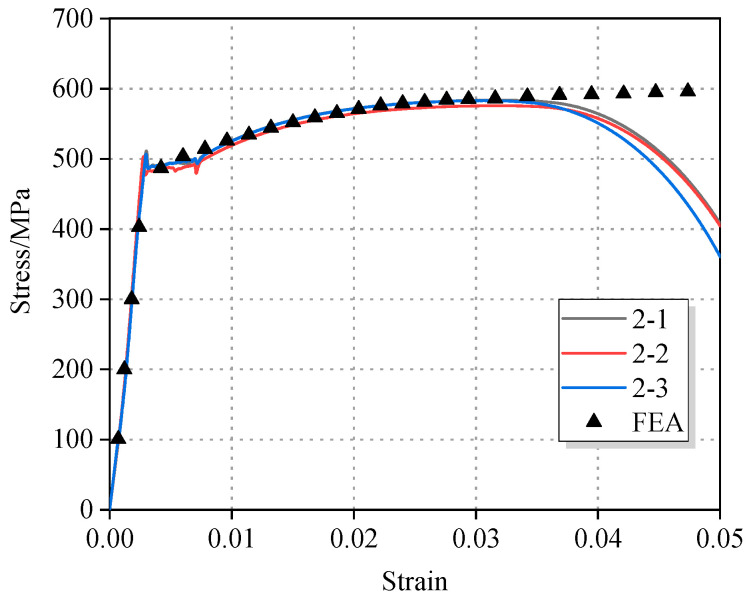
Comparison of finite element simulation results based on the Johnson–Cook constitutive model and tensile test data.

The comparison between the finite element (FE) simulated stress–strain curve based on the Johnson–Cook constitutive model and the experimental stress–strain curve is shown in Figure 5. Before the material reached its ultimate tensile strength and necking occurred, the FE simulation exhibited a high degree of agreement with the experimental data. The maximum deviation between the finite element results and the experimental data curves in the elastic stage, yield plateau, and strain hardening phase came out to less than 2%.

(5) $$\bar{\sigma }=450+660.92{\left({\bar{\varepsilon }}_{p}\right)}^{0.4482}$$

## 3. Prediction of Failure Pressure

### 3.1. Finite Element Model

In this study, a finite element model of a defective pipeline was developed using ANSYS software R2 (v. 22.2.0). The model employed SOLID186 hexahedral elements as the basic computational units. Internal corrosion of the pipeline is primarily caused by electrochemical reactions, with pitting and localized corrosion being the most common forms. Based on this, the geometry of the corroded surface was simplified to a regular rectangular shape, and the corrosion depth was assumed to be a uniform reduction in wall thickness. This simplification served as the foundation for building the 3D finite element model of the corroded pipeline. According to existing studies [24], the geometric shape of the defect edges has a negligible effect on the pipeline’s burst pressure; therefore, sharp-edge treatment was adopted in this study.

**Figure 6:**
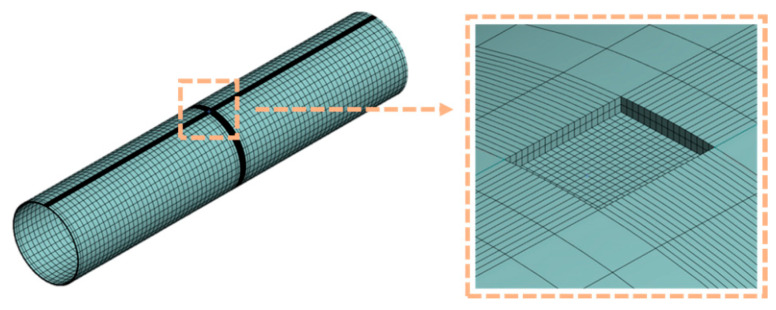
Finite element model of defective pipes.

During normal operation, the pipeline is mainly subjected to internal pressure, which generates hoop stress, causing radial expansion. Given that the pipeline length is much greater than its diameter and both ends are open, the effect of axial displacement is negligible. Therefore, axial displacement constraints were applied at both ends of the pipeline model to restrict axial movement. To minimize the influence of Poisson’s effect on the simulation results, the model length was set to five times the outer diameter [25], and mesh refinement was applied in the defect region. The finite element model of the corroded pipeline is shown in Figure 6.

A plastic failure criterion was adopted to evaluate pipeline failure. That is, when the Von Mises equivalent stress in the corroded region reaches the ultimate tensile strength of the pipe material, the pipeline is considered to have failed [25]. The corresponding internal pressure at this point is defined as the failure pressure.

Ensuring the accuracy of the finite element model is a prerequisite for reliable numerical analysis. To validate the model, three representative pipelines with external surface defects from the literature [26] were selected. These pipelines were all made of X80-grade steel, with an outer diameter of 458.8 mm and a wall thickness of 8.1 mm. The geometric parameters of the corrosion defects for each pipeline are listed in Table 3.

**Figure 7:**
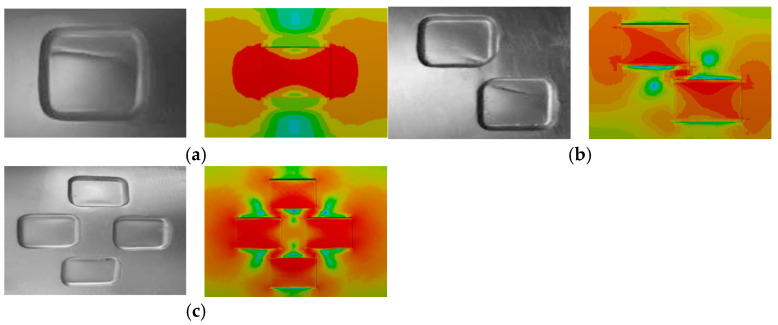
Comparison of blasting test results and finite element analysis results: (**a**) ITDS-2; (**b**) ITDS-5; (**c**) ITDS-6.

The finite element validation results are presented in Table 4. The relative error between the calculated results of the present model and the experimental data ranged from 3.6% to 8.6%, with an average error of 5.6%. As shown in Figure 7, a comparison of the equivalent stress distribution at failure and the actual rupture locations observed in the burst tests indicates a high degree of agreement, verifying the rationality of the model’s boundary conditions and loading setup. In addition, the simulated failure pressures closely match the experimental values, demonstrating that the model can accurately predict the burst pressure of corroded pipelines. Therefore, the validated finite element approach was employed in this study to construct a failure pressure dataset for pipelines with varying defect sizes and diameters, serving as the foundation for predictive modeling of corrosion-defected pipeline failure.

### 3.2. GABP Neural Network Model

**Figure 8:**
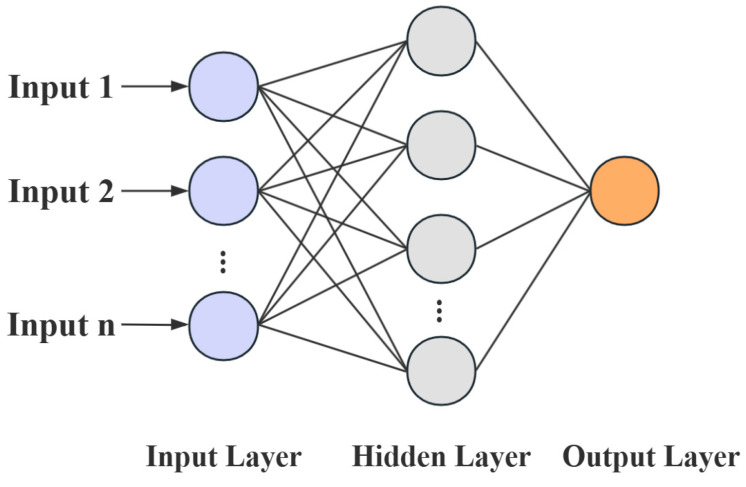
BP neural network structure diagram.

The architecture of the BP neural network, as illustrated in Figure 8, consists of an input layer, one or more hidden layers, and an output layer. The layers are interconnected through weight matrices and threshold vectors, with each node in the hidden and output layers assigned a specific threshold value. The learning process of the network primarily involves forward signal propagation, backward error propagation, and iterative optimization of weights and thresholds to minimize the prediction error.

However, in practical applications, the BP neural network model often suffers from poor prediction performance due to improper initialization of weights and biases, which may cause the model to fall into a local optimum. To enhance the predictive accuracy of the BP neural network, this study employed a genetic algorithm (GA) to optimize the network [27,28], thereby achieving more accurate predictions of burst pressure and residual service life.

The core mechanism of genetic optimization lies in the selection, crossover, and mutation operations performed on individuals based on a defined fitness function, allowing high-fitness offspring to be retained. These offspring inherit the primary characteristics of their parents while introducing new variations. During the evolutionary process, beneficial traits that better adapt to the environment are preserved, while less suitable traits are gradually eliminated. Through this iterative optimization process, the population ultimately converges toward the optimal solution.

**Figure 9:**
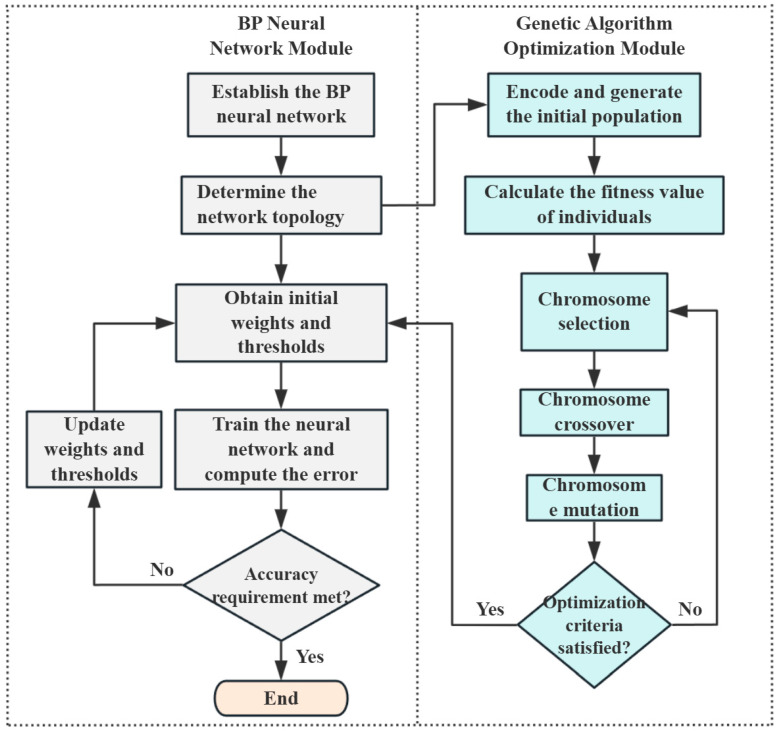
Flowchart of genetic algorithm optimization of BP neural network.

In the context of BP neural network optimization, the objective is to refine the connection weights and biases between the input layer and hidden layer, as well as between the hidden layer and output layer. Initially, the network’s weights and thresholds are encoded as chromosomes in the genetic algorithm, and a population is randomly initialized. The mean squared error (MSE) of the BP network is used as the fitness function. Individual fitness values are then calculated to guide the evolutionary process. If the current parameter set has not reached optimal performance, iterative genetic operations—selection, crossover, and mutation—are applied to evolve the population until an optimal parameter combination is obtained. These optimized parameters are then imported into the BP network, completing the GA-based optimization. The optimization flow is illustrated in Figure 9.

The training data for the BP neural network in this study were obtained from two sources: experimental burst test data of defective pipelines reported in the literature [26,29,30,31,32,33,34,35] and the dataset established in Section 3.1 of this study. A total of 270 data samples were compiled, as detailed in Appendix A. Fifteen representative models were selected and retained as comparative samples for evaluating failure pressure prediction between the GABP neural network and existing assessment methods and were not included in the neural network training process. The remaining 255 data samples were randomly divided into a training set (70%), a validation set (20%), and a testing set (10%).

The defect parameters were used as input variables, with a total of ten inputs that includes the defect length (*L*), width (*w*), depth (d), axial spacing (*S_L_*), circumferential spacing (*S_C_*), internal or external position (*P*), pipe diameter (*D*), wall thickness (*t*), yield strength (*σ_y_*), and ultimate tensile strength (*σ_u_*). The failure pressure (*P_f_*) was taken as the output variable. For the hidden layer, the number of neurons plays a critical role. An insufficient number of neurons may result in underfitting, failing to capture the complex nonlinear relationships, while an excessive number can lead to overfitting. Therefore, an empirical formula (6) was initially adopted to estimate the number of neurons, which was subsequently fine-tuned based on the training performance to determine the optimal network configuration [36].

(6) $$h=\sqrt{p+q}+a$$

where *h* denotes the number of neurons in the hidden layer; *p* represents the number of nodes in the input layer; *q* is the number of nodes in the output layer; and *a* is an integer ranging from 1 to 10.

The training performance of the neural network can be evaluated using the mean squared error (MSE). A smaller MSE indicates higher prediction accuracy. The expression for MSE is given as [37]

(7) $$MSE=\frac{1}{n}{\sum_{i=1}^{n}\left(Y_{i}-Y_{i}^{\prime }\right)}^{2}$$

where *n* denotes the number of input samples, *Y*_1_ represents the actual value, and $Y_{i}^{\prime }$ refers to the predicted value generated by the model.

**Figure 10:**
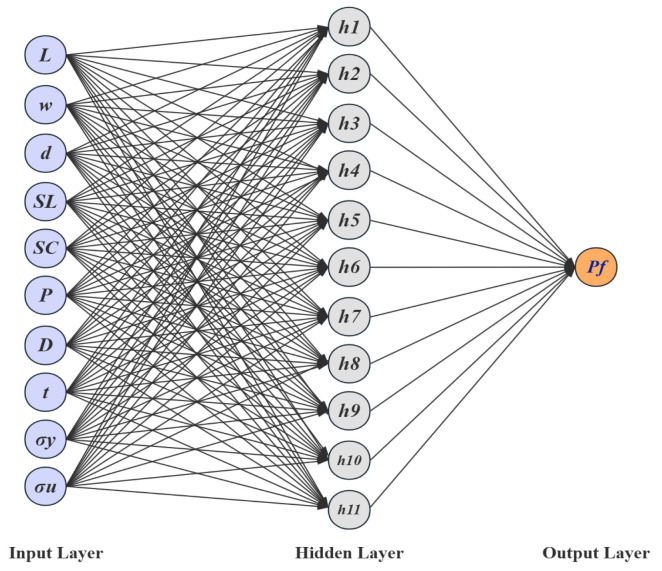
The BP neural network structure for predicting failure pressure of defective pipelines.

**Figure 11:**
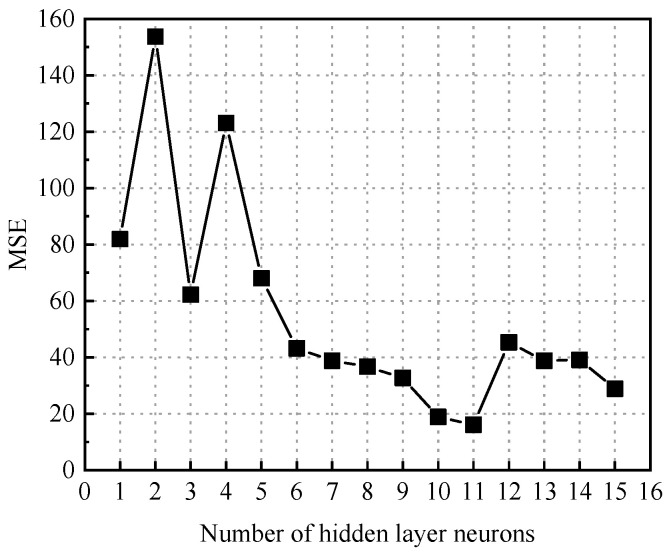
The MSE values associated with varying numbers of hidden layer neurons in the neural network model employed for failure pressure prediction.

The structure of the BP neural network for predicting the failure pressure of defective pipelines is shown in Figure 10. The training process of the GABP neural network for predicting the failure pressure of corroded pipelines included the following steps: numerical initialization, preliminary training of the BP neural network, optimization of the network weights and thresholds using a genetic algorithm, retraining of the BP neural network with the optimized parameters, and finally, the construction of the GABP neural network model. The genetic algorithm parameters for predicting the failure pressure of defective pipes are shown in Table 5. The entire optimization and training process was implemented using the MATLAB (R2022b) numerical simulation platform. The training dataset was fed into the GABP algorithm module, where the genetic algorithm iteratively optimized the BP neural network. During the construction of the BP neural network model, different numbers of hidden layer neurons were tested to identify the optimal configuration based on the minimum mean squared error (MSE). As shown in Figure 11, the MSE reached its lowest value when the number of hidden neurons was set to 11, indicating that this configuration yielded the best prediction performance.

**Figure 12:**
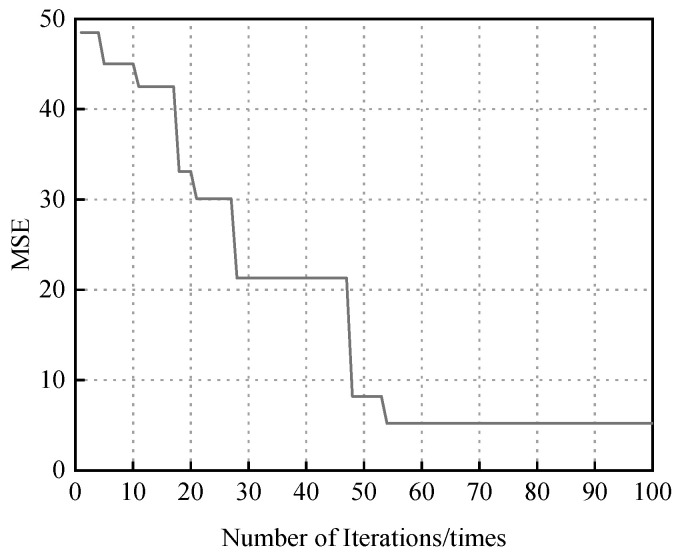
Genetic algorithm optimization of BP neural network convergence curve.

The convergence curve of the genetic algorithm optimization is shown in Figure 12. The horizontal axis represents the number of iterations, while the vertical axis represents the fitness function value, i.e., the mean squared error (MSE). As observed in the figure, the MSE value stabilized after approximately 53 generations, indicating that the genetic algorithm had converged to the optimal solution. At this point, the minimum MSE no longer changed and remained stable at a value of 5.19, confirming the effectiveness of the GA-based optimization process.

### 3.3. Optimized Results

**Figure 13:**
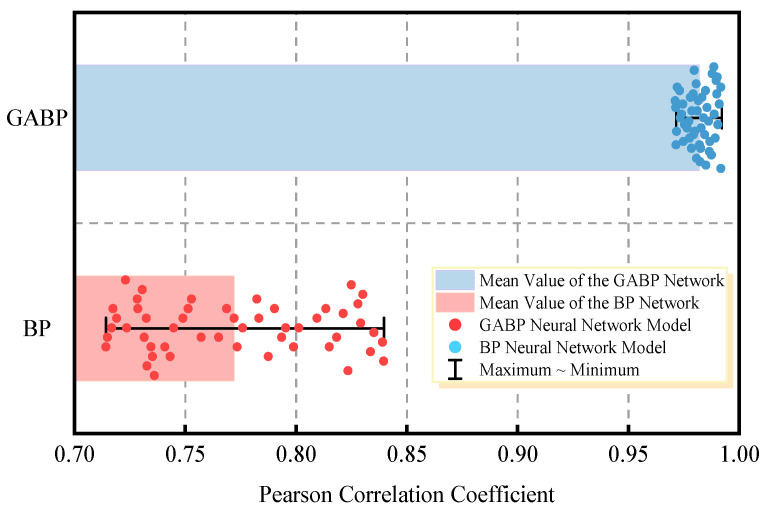
Pearson correlation coefficients corresponding to 50 independent training runs.

A total of 50 independent training sessions were conducted for both the BP and GABP neural networks. The performance of these models was evaluated using the Pearson Correlation Coefficient (R), where a value closer to 1 indicates a better fit between predicted and actual values. The Pearson coefficients for all 50 training instances are shown in Figure 13. The BP neural network yielded an average R value of 0.7689 with a standard deviation of 0.0478, whereas the GABP network achieved a significantly higher mean of 0.9824 and a lower standard deviation of 0.0196. These results suggest that the GA-BP model not only provides greater predictive accuracy but also demonstrates enhanced stability, as indicated by the narrower distribution range and higher proximity to R = 1. The Wilcoxon signed-rank test yielded a *p*-value of 0.0012, indicating that the difference in prediction performance between the two models is statistically significant at the 95% confidence level.

**Figure 14:**
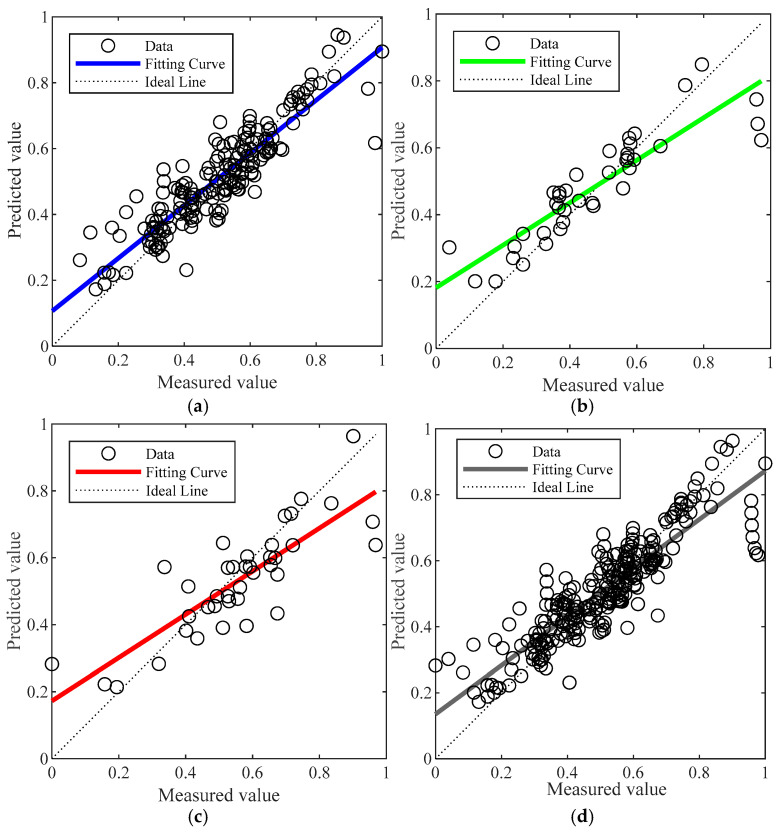
Fitting effect diagram of BP neural network: (**a**) training set fitting effect diagram; (**b**) validation set fitting effect diagram; (**c**) test set fitting effect diagram; (**d**) overall fitting effect diagram.

**Figure 15:**
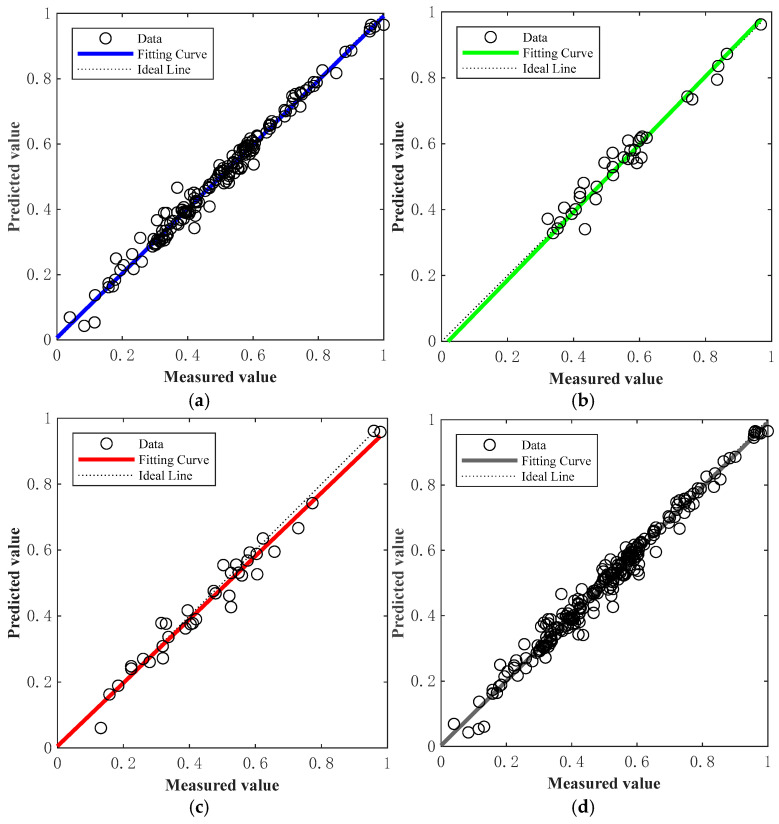
Fitting effect diagram of GABP neural network: (**a**) training set fitting effect diagram; (**b**) validation set fitting effect diagram; (**c**) test set fitting effect diagram; (**d**) overall fitting effect diagram.

Figure 14 and Figure 15 present the regression plots corresponding to the best-performing models (in terms of R value) for the training, testing, and validation datasets of the BP and GABP networks, respectively. All data were normalized prior to plotting. In these figures, the diagonal “Ideal Line” represents the condition where R = 1, indicating perfect agreement between the predicted and actual values. The closer the regression curve is to this line, the better the fitting performance of the network.

A comparison of the regression results reveals that the BP network exhibited relatively low correlation across the training (R = 0.85426), validation (R = 0.81085), and testing (R = 0.82248) sets, resulting in an overall correlation coefficient of R = 0.83993. These values reflect a less reliable predictive performance, with substantial discrepancies between the measured and predicted outcomes. In contrast, the GA-BP network—optimized by adjusting the weights and thresholds between the input and hidden layers—achieved significantly higher correlation coefficients of 0.99671 (training), 0.98098 (validation), and 0.97949 (testing), with an overall R value of 0.99251. These results are nearly ideal (R = 1), and as shown in the figures, the data points are closely clustered around the diagonal line, indicating minimal deviation and superior fitting performance. This outcome demonstrates that the GABP neural network offers a more accurate and reliable method for predicting the failure pressure of corroded pipelines.

The optimization results of the BP neural network using the genetic algorithm are represented by weight and bias matrices. By fixing these matrices during retraining, the BP neural network can consistently converge to a stable optimal solution, thereby ensuring high prediction accuracy and reproducibility. The optimized matrices corresponding to the best-performing model among the 50 training sessions are shown below.

The weight matrix *w_ij_* from the hidden layer to the output layer is given as follows:

(8) $$\left[\begin{array}{cccccccccc} 1.145 & -2.981 & 0.718 & 0.302 & 0.261 & -1.825 & 0.526 & 0.307 & -0.847 & -0.130\\ 0.310 & -1.070 & 0.254 & 1.746 & -1.661 & -0.401 & 0.541 & 0.527 & -1.184 & -2.191\\ -1.375 & 0.683 & 1.587 & 1938 & 0.113 & -0.119 & -2.454 & 0.730 & 0.063 & -1.367\\ -0.260 & -0.376 & 1.873 & -0.065 & -0.223 & 1.128 & -1.089 & -1.007 & 1.097 & 0.256\\ 0.862 & -0.507 & 1.143 & -3.142 & 0.666 & -0.913 & -2.532 & 0.252 & 2.671 & -1.219\\ -0.734 & -1.938 & 0.362 & 1.170 & -1.383 & -0.368 & -1.401 & -0.274 & 1.533 & 0.592\\ -1.193 & 1.157 & -0.236 & 0.448 & -0.438 & -2.626 & -0.956 & -0.032 & 0.136 & -1.391\\ 0.384 & -0.578 & -2.016 & -0.221 & -1.905 & -0.251 & 0.210 & 1.765 & -1.136 & -0.145\\ -1.404 & -0.473 & 2.493 & -0.513 & -1.291 & 0.237 & 0.651 & 1.936 & 0.311 & -0.348\\ 0.088 & -0.146 & 0.788 & 1.574 & -0.936 & -0.936 & -0.413 & 0.881 & -0.329 & 1.459\\ 0.155 & -0.038 & -0.270 & 0.692 & 1.750 & -0.145 & 1.660 & -0.311 & -1.70 & -0.955 \end{array}\right]$$

The weight matrix *w_jk_* from the hidden layer to the output layer is given as follows:

(9) $${\left[-0.667\;-0.5765\;-0.303\;0.502\;0.127\;0.131\;0.748\;0.658\;0.137\;0.068\;0.362\right]}^{T}$$

The bias matrix *b_j_* for the hidden layer neurons is given as follows:

(10) [2.938 0.281 0.432 0.061 0.845 0.425 −0.955 −2.638 0.251 1.049 0.830 ]

The bias matrix *b_k_* for the output layer neurons is given as follows:

(11) $$b_{k}=\left[1.152\right]$$

In the BP neural network, the weighted sum of input parameters plays a crucial role in feature extraction, importance evaluation, nonlinear mapping, and error propagation. This value indicates the degree of influence each input parameter has on the output within the GABP neural network. A larger value signifies that the activation function primarily adjusts the output based on that particular feature. The calculation formula for the weighted sum of input parameters is given by [38]

(12) $$z=\sum_{i=1}^{n}w_{i}x_{i}+b$$

where *z* represents the weighted sum; *x_i_* denotes the value of the *i*-th input feature; *w_i_* is the corresponding weight; *b* is the bias term; and *n* is the total number of input parameters.

**Figure 16:**
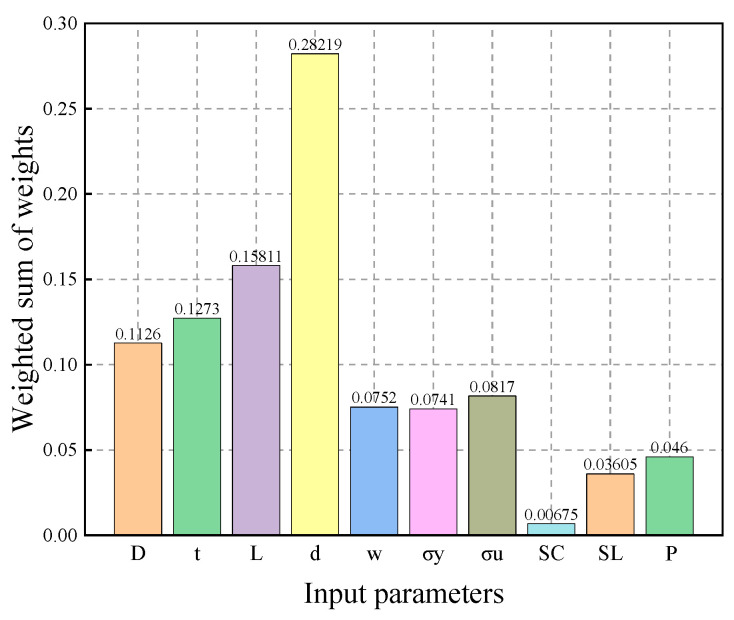
The impact of the weighted summation of individual parameter weights on the prediction of failure pressure in the neural network model.

The weighted sums of the input parameters in the GABP neural network are shown in Figure 16. Among these parameters, defect depth d was found to have the greatest influence on failure pressure, while circumferential spacing Sc had the least. This result indicates that the defect depth is the most critical parameter in predicting the failure pressure of defective pipelines using the neural network, and accurately determining the defect depth is essential for the reliability of the assessment.

### 3.4. Model Validation

**Figure 17:**
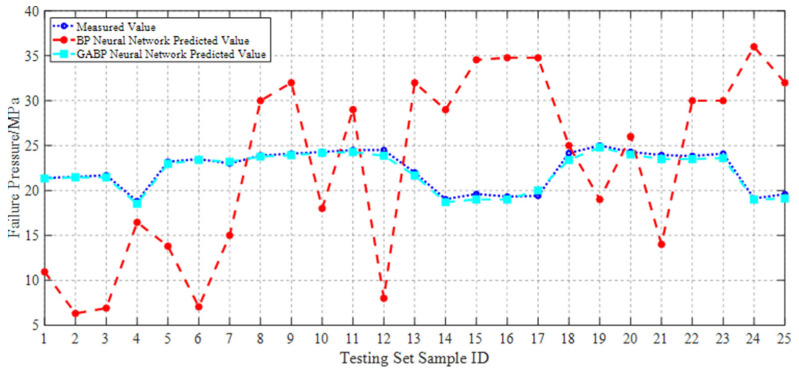
Comparison of GABP and BP neural network prediction results.

During the training and optimization processes of both the BP and GABP neural networks, the trends of the measured values, BP-predicted values, and GABP-predicted values for each sample in the testing set are illustrated in Figure 17. It can be observed that the BP neural network exhibits significant fluctuations in its predictions, with error distributions lacking clear regularity.

**Figure 18:**
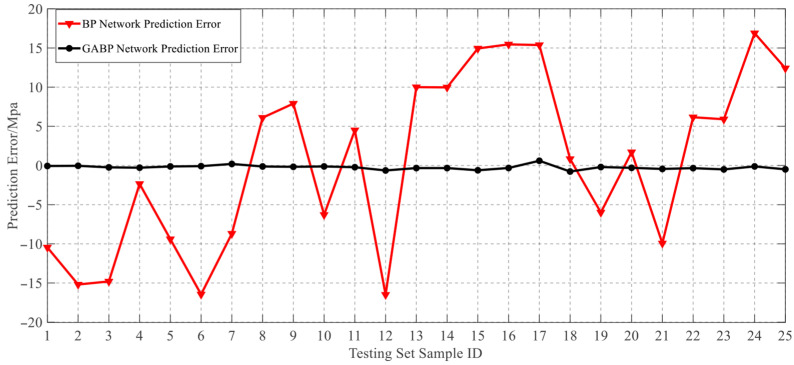
Comparison of GABP and BP neural network prediction errors.

In contrast, the GABP neural network demonstrated a substantial improvement in prediction accuracy. The maximum deviation between the predicted and actual failure pressures by the GABP model was 2.3 MPa, corresponding to a relative error of 9%. Furthermore, as shown in the error comparison plot in Figure 18, the prediction deviations of the GABP neural network consistently fluctuated around zero, indicating that the BP neural network optimized via a genetic algorithm achieved superior accuracy in predicting the failure pressure of corroded pipelines. In this context, the prediction error was defined as the neural network output minus the experimentally measured value.

**Figure 19:**
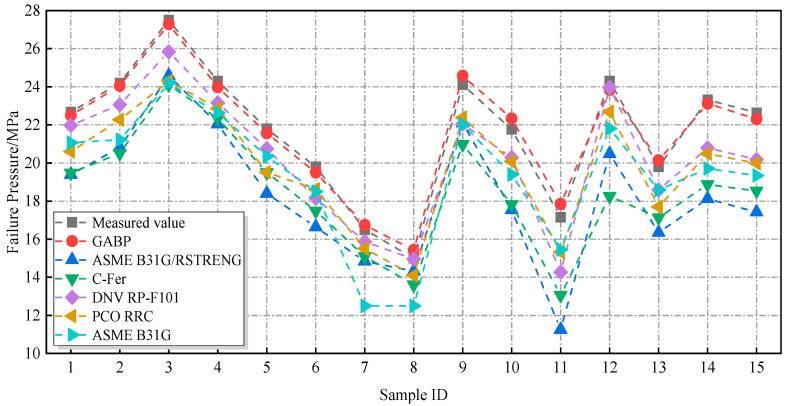
Comparison of failure stresses predicted by various evaluation methods and the GABP network.

The 15 sets of burst test data for defective pipelines retained in Section 3.2, which were not involved in the training process of the neural network, were selected to validate the accuracy and generalization capability of the GABP neural network model. The defect parameters of these pipelines are summarized in Table 6. These parameters were input into the trained GABP network to generate predicted failure pressures. In parallel, failure pressures were also calculated using conventional standards, including DNV RP-F101, PCORRC, ASME B31G, ASME B31G/RSTRENG, and C-FER, for comparison. As illustrated in Figure 19, the results indicate that the failure pressures estimated by traditional assessment methods were generally lower than the actual measured values, reflecting a conservative bias. In contrast, the predictions produced by the GA-BP neural network showed strong agreement with the experimental results, demonstrating high prediction accuracy. The maximum deviation between predicted and actual failure pressures was only 4.08%.

## 4. Prediction of Remaining Life of Pipelines

### 4.1. Corrosion Rate Prediction Model

In high-sulfur-containing corrosive environments, the corrosion rate of X52 steel is influenced by multiple factors, primarily including the partial pressures of CO_2_ and H_2_S, the temperature of the transported medium, and the flow velocity. These factors exhibit a highly nonlinear and complex mapping relationship with the corrosion rate rather than a simple linear correlation [39,40]. Currently, comprehensive mathematical models that consider multiple corrosion factors remain insufficiently developed. However, artificial neural networks, due to their powerful fuzzy recognition and nonlinear prediction capabilities, provide an effective approach for corrosion rate prediction. Therefore, in this study, a corrosion rate prediction model based on a genetic algorithm (GA)-optimized BP neural network was constructed to enhance prediction accuracy and adaptability, thereby enabling a more precise assessment of the corrosion behavior of X52 steel in high-sulfur environments. The genetic algorithm parameters used to predict the sulfur corrosion rate are shown in Table 7.

**Figure 20:**
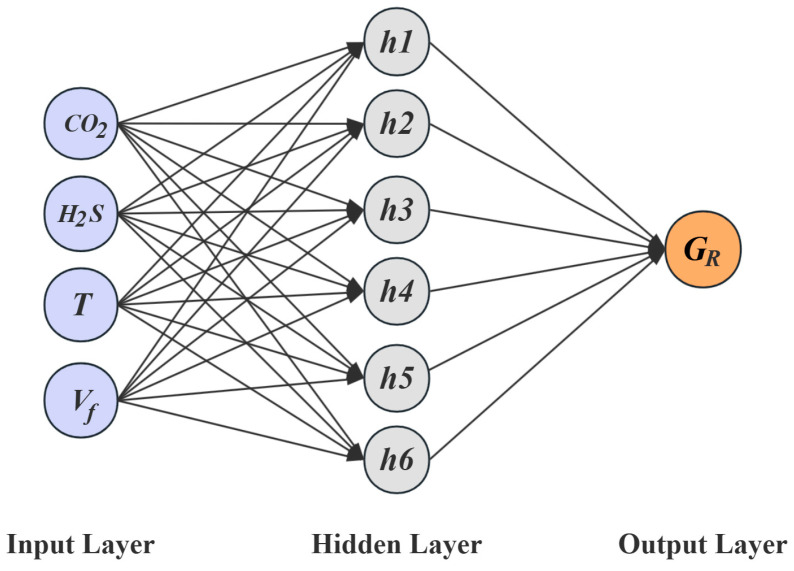
BP neural network structure diagram for predicting sulfur corrosion rates.

**Figure 21:**
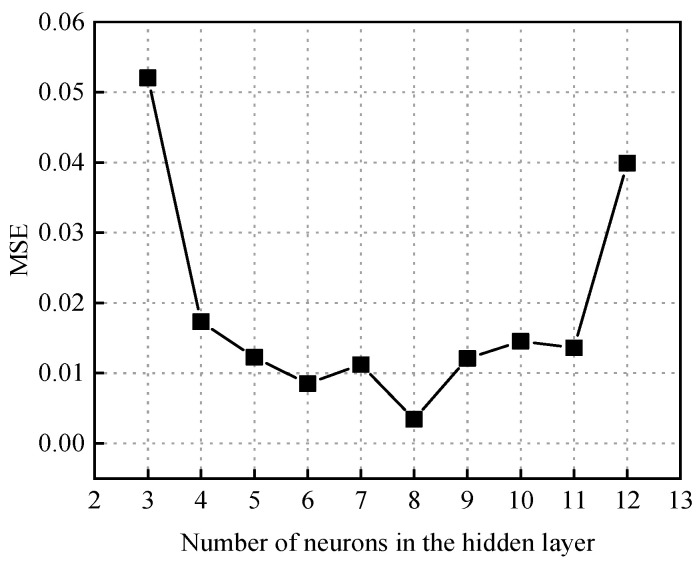
The MSE values associated with varying numbers of hidden layer neurons in the neural network model developed for predicting the sulfur corrosion rate.

**Figure 22:**
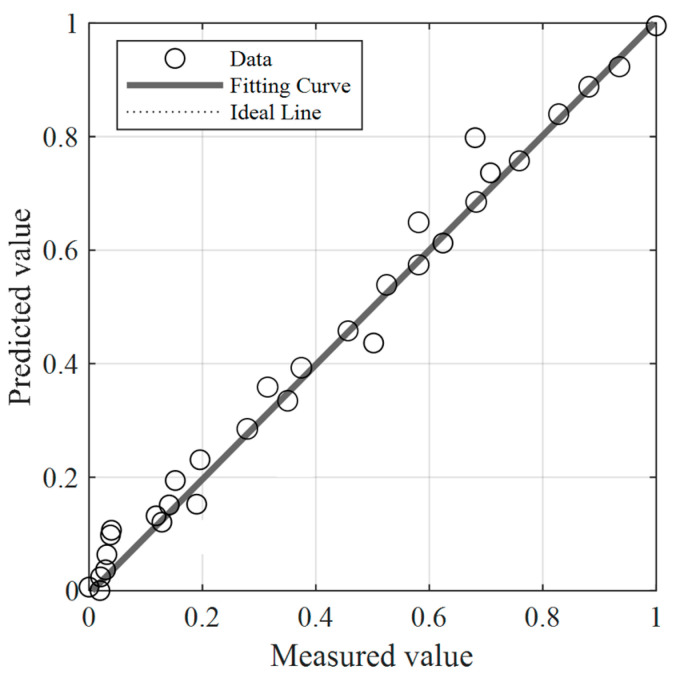
GABP network prediction corrosion rate fitting diagram.

The existing literature [39,41,42] indicates that the primary factors affecting the corrosion rate of pipelines in CO_2_ and H_2_S coexisting environments are the partial pressures of corrosive substances, the temperature of the transported medium, and the flow velocity of the transported medium. Accordingly, these parameters were selected as the input layer of the neural network, comprising four input variables, with the corrosion rate as the output layer, having one output target. The BP neural network structure for predicting the sulfur corrosion rate is shown in Figure 20. The validation results show that with four inputs and one output, setting the number of neurons in the hidden layer to eight yielded the minimum mean squared error (MSE), and the MSE values corresponding to different numbers of hidden layer neurons are presented in Figure 21. Thirty samples of X52 steel corrosion rates related to sulfur-containing corrosion were collected from references [43,44,45,46,47] as the training data. The training results, shown in Figure 22, demonstrate that the GABP network model achieved an MSE of 0.0369 and a correlation coefficient of 0.99544.

**Figure 23:**
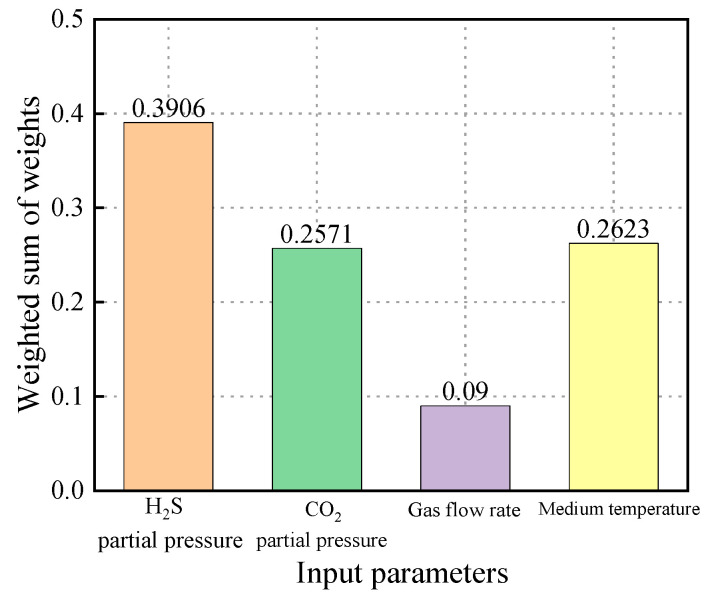
The impact of the weighted summation of individual parameter weights on the prediction of sulfur corrosion rate within the neural network model.

The weighted sums of the input parameters are shown in Figure 23. The results indicate that the partial pressure of H_2_S had the greatest impact on the prediction outcome, with a weighted sum of 0.3906. The influences of CO_2_ partial pressure and the flow velocity of the transported medium were approximately equal, with weighted sums of 0.2571 and 0.2623, respectively. In contrast, temperature had the least effect on the prediction results, with a weighted sum of 0.09.

The optimized weight matrix *w_ij_* from the input layer to the hidden layer is as follows:

(13) $$\left[\begin{array}{l} 2.153\;0.170\;1.826\;0.198\\ 1.499\;1.611\;2.231\;0.337\\ 1.427\;0.240\;0.061\;1.354\\ 2.154\;1.255\;2.042\;2.025\\ 1.720\;0.634\;2.620\;0.693\\ 0.655\;0.631\;1.678\;1.979\\ 1.266\;1.731\;1.450\;1.335\\ 2.480\;0.402\;1.201\;3.017 \end{array}\right]$$

The weight matrix *w_jk_* from the hidden layer to the output layer is as follows:

(14) $${\left[\;0.375\;0.936\;0.801\;0.945\;0.985\;0.456\;0.731\;0.593\right]}^{T}$$

The bias matrix *b_j_* for the hidden layer neurons is as follows:

(15) [2.511 1.847 1.249 2.216 0.910 1.623 2.738 2.636]

The bias matrix *b_k_* for the output layer neurons is as follows:

(16) $$b_{k}=\left[1.324\right]$$

The correlation coefficient and mean squared error (MSE) of the network indicate a high degree of fit, demonstrating that this neural network is well suited for predicting the corrosion rate of X52 steel in sulfur-containing environments. Validation was performed using six datasets from reference [43], with the results presented in Table 8. The maximum prediction error was found to be 7.94%. These results confirm that the model can accurately predict the corrosion rate of X52 steel under the combined effects of H_2_S and CO_2_, highlighting its significant engineering applicability.

### 4.2. Remaining Life Prediction Method

Based on the method for determining the remaining life of corroded pipelines proposed in the American standard NACE SP 0502-2010 “Direct Assessment Method for Pipeline Corrosion” [48], the remaining life can be predicted according to the pipeline operating pressure, wall thickness, and corrosion rate. The expression is given as follows:

(17) $$T_{R}=C\times S_{M}\frac{t}{G_{R}}$$

where *C* represents the correction factor and is taken as 0.85; *t* denotes the wall thickness of the pipeline; *G_R_* stands for the corrosion rate; and *S_M_* indicates the safety margin.

**Figure 24:**
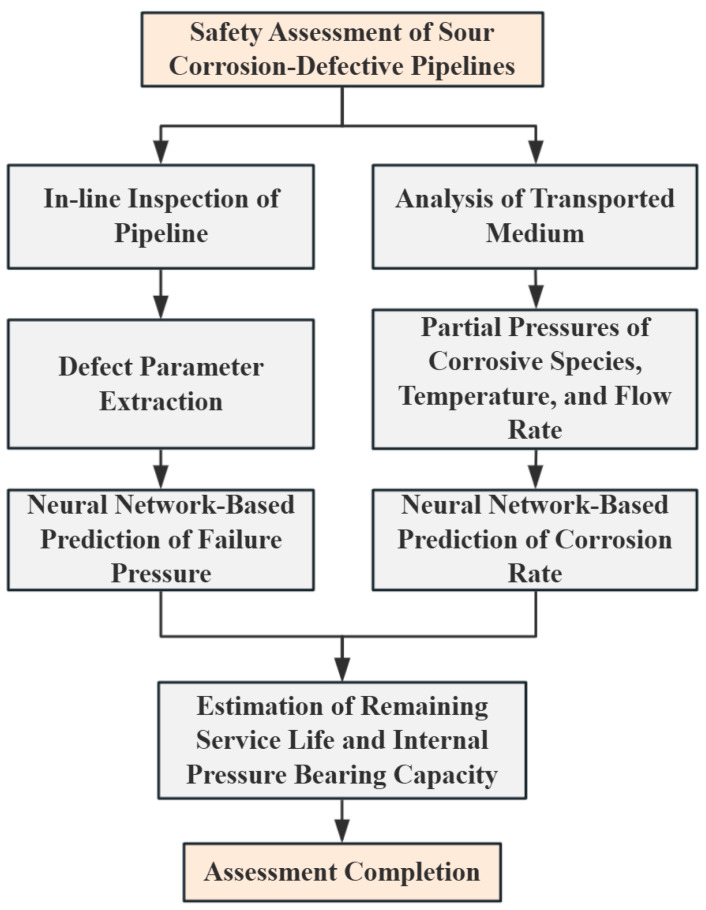
Prediction process for residual bearing capacity of pipelines with sulfur corrosion defects.

Based on the GABP network model established in this study, the failure pressure of the corrosion-defected pipeline and the defect corrosion rate under its service environment were first predicted. Subsequently, the remaining service life of the defective pipeline was calculated using Equation (17). The entire process was implemented through programming on the Matlab platform, where the pre-trained GABP networks for failure pressure and corrosion rate prediction were integrated with the remaining life prediction formula. By inputting the relevant parameters, predictions and evaluations of both the failure pressure and remaining life could be achieved. The detailed procedure is illustrated in Figure 24.

### 4.3. Model Application Illustrative Example

Taking a segment of a sulfur-containing gas field’s gathering pipeline as an example, the pipeline material examined was X52 steel, with a diameter of 323 mm and a wall thickness of 13 mm. The design operating pressure was set to 9.6 MPa, while the actual operating pressure ranged from 5.2 to 8.2 MPa. The transported medium temperature was set to 30 °C, with partial pressures of H_2_S and CO_2_ measured at 0.116 MPa and 0.126 MPa, respectively. The gas flow velocity was set to 4 m/s. Coupon tests determined the corrosion rate of this pipeline segment to be 0.1 mm/year, while the corrosion rate predicted by the neural network was 0.098 mm/year. In-line inspection results revealed the presence of five independent corrosion defects within the pipeline segment. The corrosion defects were sufficiently spaced to ensure that their impacts on the remaining service life could be regarded as independent. Based on this assumption, the five defects were treated as independent failure sites during the remaining life evaluation. By inputting these parameters into the prediction model, the failure pressure and remaining service life of the pipeline segment were obtained. The defect parameters, failure pressures, and remaining service lives are summarized in Table 9. Without any protective measures, the minimum failure pressure of this segment was predicted to be 22.3 MPa, which satisfies the design pressure requirement of 9.6 MPa. However, the minimum remaining service life was found to be only 4.6 years, indicating a need for enhanced monitoring and timely replacement of this pipeline segment.

## 5. Conclusions

(1) The mechanical properties of pipeline steel exposed to high-sulfur corrosive environments were experimentally investigated to assess the effects of such harsh service conditions on material strength degradation. The results indicated that, despite prolonged exposure to a high-sulfur environment, the material property parameters of the corroded pipeline steel exhibited no significant degradation in its intrinsic mechanical properties, although geometric stability was reduced due to corrosion.
(2) The Johnson–Cook constitutive model for X52 pipeline steel was calibrated, with the numerical simulation results showing a high degree of agreement with the experimental curves. This constitutive model was demonstrated to accurately describe the plastic hardening characteristics of X52 steel.
(3) A genetic algorithm-optimized backpropagation (GABP) neural network model was developed to predict the failure pressure and corrosion rate of defected pipelines. The predictive accuracy and fitting performance of the GABP model were compared with those of the conventional BP neural network. The results show that the GABP neural network achieved higher prediction accuracy for both the pipeline failure pressure and X52 sulfur corrosion rate, with maximum errors of 4.08% and 7.94%, respectively. Furthermore, based on the two neural network models, the failure pressure and service life of an in-service pipeline in a sulfur-containing gas field were predicted, demonstrating strong practical engineering guidance value.

## Figures and Tables

**Table 1:** Pipe dimensions and specimen numbers.

Pipe Number	#1	#2	#3
Diameter (mm)	323	323	273
Wall thickness (mm)	13	13	11
Test piece number	1-1, 1-2, 1-3	2-1, 2-2, 2-3	3-1, 3-2, 3-3

**Table 2:** Results of material property tests on each test piece.

Test Specimen Number	*σ_y_*/MPa	*σ_u_*/MPa	*E*/MPa
1-1	432	479	205,216
1-2	420	509	206,195
1-3	457	550	205,958
Average value of pipe #1	436.3	512	206,123
2-1	490	583	205,598
2-2	469	566	205,412
2-3	489	582	206,427
Average value of pipe #2	482	577	205,812
3-1	431	551	206,572
3-2	410	511	205,127
3-3	427	529	205,457
Average value of pipe #3	423	530	205,718

**Table 3:** Finite element verification model parameters.

Model Number	Defect Depth *d*/mm	Defect Length *L*/mm	Defect Width *w*/mm	Defect Axial Spacing *S_L_*/mm	Defect Circumferential Spacing *S_C_*/mm
IDTS-2	5.38	39.5	31.8	\	\
IDTS-5	5.42	39.5	32.1	−9.5	10
IDTS-6	5.38	39.5	32.1	20.4	9.5

**Table 4:** Finite element verification results.

Model Number	Burst Pressure (MPa)	Numerical Simulation Result (MPa)	Error (%)
IDTS-2	22.67	21.6	4.6
IDTS-5	20.87	19.2	8.6
IDTS-6	18.65	18.0	3.6

**Table 5:** Genetic algorithm parameters for predicting the failure pressure of defective pipes.

Indicator	Population Size	Chromosome Length	Maximum Generation	Crossover Rate	Mutation Rate	Number of Neurons in the Input Layer	Number of Neurons in the Hidden Layer	Number of Neurons in the Output Layer	Selection Mechanism
Value	50	133	100	0.8	0.05	10	11	1	Roulette wheel selection

**Table 6:** Pipe parameters for failure pressure prediction verification.

Serial Number	*D*/mm	*t*/mm	*L*/mm	*d*/mm	*w*/mm	Test Value/MPa	GABP Predicted Value/MPa	Error/%
1	458.8	8.10	39.60	5.39	31.90	22.68	22.5	0.79
2	459.4	8.00	40.05	3.75	32.00	24.2	24.05	0.62
3	323.9	9.80	255.60	7.08	95.30	27.5	27.29	0.76
4	323.9	9.66	305.60	6.76	95.30	24.3	23.98	1.32
5	323.9	9.71	350.00	6.93	95.30	21.8	21.57	1.06
6	323.9	9.71	394.50	6.91	95.30	19.8	19.5	1.52
7	323.9	9.91	433.40	7.31	95.30	16.5	16.74	1.45
8	323.9	9.74	466.70	7.02	95.30	15	15.43	2.87
9	323.9	9.79	488.70	6.99	95.30	24.11	24.58	1.95
10	323.9	9.79	500.00	6.99	95.30	21.76	22.33	2.62
11	323.9	9.74	527.80	7.14	95.30	17.15	17.85	4.08
12	762	17.50	50.00	8.75	50.00	24.3	23.85	1.85
13	762	17.50	100.00	8.75	50.00	19.8	20.14	1.72
14	762	17.50	200.00	8.75	50.00	23.32	23.12	0.86
15	762	17.50	300.00	8.75	50.00	22.64	22.31	1.46

**Table 7:** Genetic algorithm parameters for predicting sulfur corrosion rates.

Indicator	Population Size	Chromosome Length	Maximum Generation	Crossover Rate	Mutation Rate	Number of Neurons in the Input Layer	Number of Neurons in the Hidden Layer	Number of Neurons in the Output Layer	Selection Mechanism
Value	50	37	100	0.8	0.05	4	8	1	Roulette wheel selection

**Table 8:** Error in neural network prediction of corrosion rate.

Serial Number	CO_2_ Partial Pressure /MPa	H_2_S Partial Pressure /MPa	Temperature /°C	Flow Velocity /(m/s)	Measured Corrosion Rate /(mm/year)	Predicted Corrosion Rate /(mm/year)	Error/%
1	0	0	40	4	0.00858	0.008	6.75
2	0	0.1	60	6	0.03325	0.0312	6.16
3	0	0.2	80	8	0.3786	0.3567	5.78
4	0.1	0	60	8	0.02618	0.0241	7.94
5	0.1	0.1	80	4	0.4066	0.3985	1.99
6	0.1	0.2	40	6	0.5038	0.4938	1.98

**Table 9:** Results of safety assessment of pipelines with sulfur corrosion defects.

Serial Number	*L*/mm	*w*/mm	*d*/mm	*P_f_*/MPa	Remaining Life/Year
1	110	21	6.1	33.0	5.621087
2	95	18	5.3	31.2	5.188696
3	87	32	7	28.8	4.612174
4	80	24	8	29.5	4.780326
5	70	18	5.9	35.0	6.101522

## Data Availability

The original contributions presented in the study are included in the article, further inquiries can be directed to the corresponding author.

## Appendix A

**Table A1:** Training dataset for neural network.

Serial Number	*D*/mm	*t*/mm	*L*/mm	*d*/mm	*w*/mm	Material	*σ_y_*/MPa	*σ_u_*/MPa	*P_f_*/MPa
1	458.8	8.10	39.60	5.39	31.90	X80	601.00	684.00	22.68
2	459.4	8.00	40.05	3.75	32.00	X80	589.00	730.50	24.20
3	762	17.50	50.00	8.75	50.00	X52	495.00	565.00	27.50
4	762	17.50	100.00	8.75	50.00	X52	495.00	565.00	24.30
5	762	17.50	200.00	8.75	50.00	X52	495.00	565.00	21.80
6	762	17.50	300.00	8.75	50.00	X52	495.00	565.00	19.80
7	762	17.50	600.00	8.75	50.00	X52	495.00	565.00	16.50
8	762	17.50	900.00	8.75	50.00	X52	495.00	565.00	15.00
9	762	17.50	200.00	4.20	50.00	X52	474.10	556.60	24.11
10	762	17.50	200.00	8.90	50.00	X52	474.10	556.60	21.76
11	762	17.50	200.00	13.10	50.00	X52	474.10	556.60	17.15
12	762	17.50	100.00	8.40	50.00	X52	474.10	556.60	24.30
13	762	17.50	300.00	8.50	50.00	X52	474.10	556.60	19.80
14	762	17.50	200.00	8.40	100.00	X52	474.10	556.60	23.42
15	762	17.50	200.00	9.00	200.00	X52	474.10	556.60	22.64
25	1422.4	19.25	180.00	10.40	0.50	X100	740.00	774.00	15.35
26	1422.4	20.10	385.00	3.80	0.50	X100	795.00	840.00	20.12
27	914.4	16.40	150.00	9.00	0.50	X100	739.00	813.00	21.40
28	914.4	16.40	450.00	6.00	0.50	X100	739.00	813.00	24.02
29	324	6.40	20.70	3.23	19.30	X52	382.00	570.00	16.64
30	324	6.01	19.35	3.60	18.99	X52	382.00	570.00	16.22
31	324	6.30	19.80	3.57	19.31	X52	373.00	522.00	15.95
32	324	6.31	20.13	3.73	19.74	X52	373.00	522.00	14.16
33	324	6.16	20.12	3.73	19.99	X52	356.00	514.00	18.85
34	324	6.27	19.91	3.73	20.20	X52	356.00	514.00	19.13
35	324	6.25	19.93	3.76	20.11	X52	356.00	514.00	19.27
36	324	6.18	19.92	3.79	20.01	X52	421.00	520.00	19.44
37	508	7.10	40.00	1.07	15.95	X70	485.00	590.00	19.02
38	508	8.70	80.00	2.61	31.90	X70	485.00	590.00	20.78
39	508	10.00	150.00	4.50	47.85	X70	485.00	590.00	19.54
40	508	12.00	300.00	7.20	63.80	X70	485.00	590.00	16.36
41	508	17.50	600.00	13.13	79.76	X70	485.00	590.00	13.79
42	660	7.10	80.00	3.20	82.90	X70	485.00	590.00	11.75
43	660	8.70	150.00	5.22	207.24	X70	485.00	590.00	11.13
44	660	10.00	300.00	7.50	20.72	X70	485.00	590.00	7.55
45	660	12.00	600.00	1.80	41.45	X70	485.00	590.00	22.67
46	660	17.50	40.00	5.25	62.17	X70	485.00	590.00	34.41
47	711	7.10	150.00	5.33	44.65	X70	485.00	590.00	6.54
48	711	8.70	300.00	1.31	66.98	X70	485.00	590.00	15.54
49	711	10.00	600.00	3.00	89.30	X70	485.00	590.00	14.54
50	711	12.00	41.00	5.40	111.63	X70	485.00	590.00	20.35
51	711	17.50	80.00	10.50	22.33	X70	485.00	590.00	25.90
52	813	7.10	300.00	2.13	127.64	X70	485.00	590.00	9.41
53	813	8.70	600.00	3.92	25.53	X70	485.00	590.00	8.79
54	813	10.00	130.00	6.00	51.06	X70	485.00	590.00	13.72
55	813	12.00	80.00	9.00	76.58	X70	485.00	590.00	13.51
56	813	17.50	150.00	2.63	102.11	X70	485.00	590.00	28.71
57	1016	7.10	600.00	4.26	95.71	X70	485.00	590.00	4.24
58	1016	8.70	40.00	6.53	127.61	X70	485.00	590.00	8.81
59	1016	10.00	80.00	1.50	159.51	X70	485.00	590.00	13.33
60	1016	12.00	150.00	3.60	31.90	X70	485.00	590.00	14.18
61	1016	17.50	300.00	7.88	63.80	X70	485.00	590.00	16.89
62	762	17.50	10.00	3.50	132.93	X52	495.00	565.00	31.40
63	762	17.50	20.00	3.50	132.93	X52	495.00	565.00	30.30
64	762	17.50	50.00	3.50	132.93	X52	495.00	565.00	29.50
65	762	17.50	100.00	3.50	132.93	X52	495.00	565.00	27.90
66	762	17.50	200.00	3.50	132.93	X52	495.00	565.00	27.50
67	762	17.50	400.00	3.50	132.93	X52	495.00	565.00	27.10
68	762	17.50	600.00	3.50	132.93	X52	495.00	565.00	26.80
69	762	17.50	800.00	3.50	132.93	X52	495.00	565.00	26.70
70	762	17.50	1000.00	3.50	132.93	X52	495.00	565.00	26.66
71	762	17.50	1200.00	3.50	132.93	X52	495.00	565.00	26.65
72	762	17.50	10.00	8.75	132.93	X52	495.00	565.00	27.90
73	762	17.50	20.00	8.75	132.93	X52	495.00	565.00	26.66
74	762	17.50	50.00	8.75	132.93	X52	495.00	565.00	25.18
75	762	17.50	100.00	8.75	132.93	X52	495.00	565.00	22.94
76	762	17.50	200.00	8.75	132.93	X52	495.00	565.00	22.42
77	762	17.50	400.00	8.75	132.93	X52	495.00	565.00	22.31
78	762	17.50	600.00	8.75	132.93	X52	495.00	565.00	21.95
79	762	17.50	800.00	8.75	132.93	X52	495.00	565.00	21.76
80	762	17.50	1000.00	8.75	132.93	X52	495.00	565.00	21.73
81	762	17.50	1200.00	8.75	132.93	X52	495.00	565.00	21.72
82	762	17.50	10.00	14.00	132.93	X52	495.00	565.00	25.20
83	762	17.50	20.00	14.00	132.93	X52	495.00	565.00	23.90
84	762	17.50	50.00	14.00	132.93	X52	495.00	565.00	22.36
85	762	17.50	100.00	14.00	132.93	X52	495.00	565.00	19.87
86	762	17.50	200.00	14.00	132.93	X52	495.00	565.00	16.88
87	762	17.50	400.00	14.00	132.93	X52	495.00	565.00	16.79
88	762	17.50	600.00	14.00	132.93	X52	495.00	565.00	16.77
89	762	17.50	800.00	14.00	132.93	X52	495.00	565.00	16.78
90	762	17.50	1000.00	14.00	132.93	X52	495.00	565.00	16.76
91	762	17.50	1200.00	14.00	132.93	X52	495.00	565.00	16.75
92	762	17.50	50.00	2.00	132.93	X52	495.00	565.00	30.86
93	762	17.50	50.00	5.00	132.93	X52	495.00	565.00	28.17
94	762	17.50	50.00	9.00	132.93	X52	495.00	565.00	25.30
95	762	17.50	50.00	14.00	132.93	X52	495.00	565.00	22.35
96	762	17.50	150.00	2.00	132.93	X52	495.00	565.00	30.00
97	762	17.50	150.00	5.00	132.93	X52	495.00	565.00	25.75
98	762	17.50	150.00	9.00	132.93	X52	495.00	565.00	22.26
99	762	17.50	150.00	14.00	132.93	X52	495.00	565.00	16.93
100	762	17.50	300.00	2.00	132.93	X52	495.00	565.00	29.40
101	762	17.50	300.00	5.00	132.93	X52	495.00	565.00	26.20
102	762	17.50	300.00	9.00	132.93	X52	495.00	565.00	22.41
103	762	17.50	300.00	14.00	132.93	X52	495.00	565.00	17.22
104	762	17.50	200.00	3.50	20.00	X52	495.00	565.00	27.50
105	762	17.50	200.00	3.50	60.00	X52	495.00	565.00	27.16
106	762	17.50	200.00	3.50	100.00	X52	495.00	565.00	26.21
107	762	17.50	200.00	3.50	140.00	X52	495.00	565.00	26.04
108	762	17.50	200.00	3.50	180.00	X52	495.00	565.00	25.93
109	762	17.50	200.00	8.75	20.00	X52	495.00	565.00	22.42
110	762	17.50	200.00	8.75	60.00	X52	495.00	565.00	21.43
111	762	17.50	200.00	8.75	100.00	X52	495.00	565.00	19.77
112	762	17.50	200.00	8.75	140.00	X52	495.00	565.00	19.29
113	762	17.50	200.00	8.75	180.00	X52	495.00	565.00	19.12
114	762	17.50	200.00	14.00	20.00	X52	495.00	565.00	16.88
115	762	17.50	200.00	14.00	60.00	X52	495.00	565.00	15.67
116	762	17.50	200.00	14.00	100.00	X52	495.00	565.00	13.97
117	762	17.50	200.00	14.00	140.00	X52	495.00	565.00	13.58
118	762	17.50	200.00	14.00	180.00	X52	495.00	565.00	13.31
119	610	12.70	17.60	6.35	47.00	X52	460.00	555.00	23.75
120	610	12.70	35.20	6.35	47.00	X52	460.00	555.00	22.50
121	610	12.70	52.80	6.35	47.00	X52	460.00	555.00	21.50
122	610	12.70	70.40	6.35	47.00	X52	460.00	555.00	20.80
123	610	12.70	88.00	6.35	47.00	X52	460.00	555.00	20.00
124	610	12.70	132.00	6.35	47.00	X52	460.00	555.00	18.30
125	610	12.70	176.00	6.35	47.00	X52	460.00	555.00	17.20
126	610	12.70	220.00	6.35	47.00	X52	460.00	555.00	16.00
127	610	12.70	264.00	6.35	47.00	X52	460.00	555.00	14.80
128	610	12.70	308.00	6.35	47.00	X52	460.00	555.00	14.20
129	457	14.00	16.00	7.00	35.00	X52	460.00	555.00	20.00
130	457	14.00	32.00	7.00	35.00	X52	460.00	555.00	18.40
131	457	14.00	48.00	7.00	35.00	X52	460.00	555.00	16.40
132	457	14.00	64.00	7.00	35.00	X52	460.00	555.00	16.00
133	457	14.00	80.00	7.00	35.00	X52	460.00	555.00	15.20
134	457	14.00	120.00	7.00	35.00	X52	460.00	555.00	14.10
135	457	14.00	160.00	7.00	35.00	X52	460.00	555.00	13.20
136	457	14.00	200.00	7.00	35.00	X52	460.00	555.00	12.50
137	457	14.00	240.00	7.00	35.00	X52	460.00	555.00	11.90
138	457	14.00	280.00	7.00	35.00	X52	460.00	555.00	10.20
139	1422	14.40	28.60	7.70	112.00	X80	638.00	739.00	16.30
140	1422	14.40	57.20	7.70	112.00	X80	638.00	739.00	15.80
141	1422	14.40	85.80	7.70	112.00	X80	638.00	739.00	14.60
142	1422	14.40	114.40	7.70	112.00	X80	638.00	739.00	14.30
143	1422	14.40	143.00	7.70	112.00	X80	638.00	739.00	14.00
144	1422	14.40	214.50	7.70	112.00	X80	638.00	739.00	11.90
145	1422	14.40	286.00	7.70	112.00	X80	638.00	739.00	10.80
146	1422	14.40	357.50	7.70	112.00	X80	638.00	739.00	9.90
147	1422	14.40	429.00	7.70	112.00	X80	638.00	739.00	9.60
148	1422	14.40	500.50	7.70	112.00	X80	638.00	739.00	9.20
149	1016	14.40	24.20	7.70	80.00	X80	638.00	739.00	23.80
150	1016	14.40	48.40	7.70	80.00	X80	638.00	739.00	22.90
151	1016	14.40	72.60	7.70	80.00	X80	638.00	739.00	21.40
152	1016	14.40	96.80	7.70	80.00	X80	638.00	739.00	20.40
153	1016	14.40	121.00	7.70	80.00	X80	638.00	739.00	19.10
154	1016	14.40	181.50	7.70	80.00	X80	638.00	739.00	17.10
155	1016	14.40	242.00	7.70	80.00	X80	638.00	739.00	16.20
156	1016	14.40	302.50	7.70	80.00	X80	638.00	739.00	15.30
157	1016	14.40	363.00	7.70	80.00	X80	638.00	739.00	13.80
158	1016	14.40	423.50	7.70	80.00	X80	638.00	739.00	13.10
159	457	14.00	80.00	7.00	35.87	X52	460.00	555.00	16.20
160	457	14.00	80.00	7.00	71.75	X52	460.00	555.00	16.00
161	457	14.00	80.00	7.00	107.62	X52	460.00	555.00	15.90
162	457	14.00	80.00	7.00	143.50	X52	460.00	555.00	15.80
163	457	14.00	80.00	7.00	179.37	X52	460.00	555.00	15.60
164	457	14.00	80.00	7.00	215.25	X52	460.00	555.00	15.30
165	610	12.70	88.00	6.35	47.89	X52	460.00	555.00	21.20
166	610	12.70	88.00	6.35	95.77	X52	460.00	555.00	21.10
167	610	12.70	88.00	6.35	143.66	X52	460.00	555.00	21.00
168	610	12.70	88.00	6.35	191.54	X52	460.00	555.00	20.90
169	610	12.70	88.00	6.35	239.43	X52	460.00	555.00	20.70
170	610	12.70	88.00	6.35	287.31	X52	460.00	555.00	20.40
171	1016	14.40	121.00	7.70	79.76	X80	638.00	739.00	19.20
172	1016	14.40	121.00	7.70	159.51	X80	638.00	739.00	19.00
173	1016	14.40	121.00	7.70	239.27	X80	638.00	739.00	18.80
174	1016	14.40	121.00	7.70	319.02	X80	638.00	739.00	18.50
175	1016	14.40	121.00	7.70	398.78	X80	638.00	739.00	18.10
176	1016	14.40	121.00	7.70	478.54	X80	638.00	739.00	17.80
177	1422	14.40	143.00	7.70	111.63	X80	638.00	739.00	14.00
178	1422	14.40	143.00	7.70	223.25	X80	638.00	739.00	13.80
179	1422	14.40	143.00	7.70	334.88	X80	638.00	739.00	13.70
180	1422	14.40	143.00	7.70	446.51	X80	638.00	739.00	13.50
181	1422	14.40	143.00	7.70	558.14	X80	638.00	739.00	13.20
182	1422	14.40	143.00	7.70	669.76	X80	638.00	739.00	12.90
183	457	14.00	80.00	2.80	71.75	X52	460.00	555.00	22.00
184	457	14.00	80.00	4.20	71.75	X52	460.00	555.00	19.90
185	457	14.00	80.00	5.60	71.75	X52	460.00	555.00	18.20
186	457	14.00	80.00	7.00	71.75	X52	460.00	555.00	14.20
187	457	14.00	80.00	8.40	71.75	X52	460.00	555.00	10.80
188	457	14.00	80.00	9.80	71.75	X52	460.00	555.00	7.50
189	457	14.00	80.00	11.20	71.75	X52	460.00	555.00	4.00
190	610	12.70	88.00	2.60	95.77	X52	460.00	555.00	23.80
191	610	12.70	88.00	3.90	95.77	X52	460.00	555.00	22.00
192	610	12.70	88.00	5.20	95.77	X52	460.00	555.00	21.50
193	610	12.70	88.00	6.50	95.77	X52	460.00	555.00	20.10
194	610	12.70	88.00	7.80	95.77	X52	460.00	555.00	18.20
195	610	12.70	88.00	9.10	95.77	X52	460.00	555.00	9.50
196	610	12.70	88.00	10.40	95.77	X52	460.00	555.00	5.20
197	1016	14.40	121.00	2.88	159.51	X80	638.00	739.00	24.00
198	1016	14.40	121.00	4.32	159.51	X80	638.00	739.00	21.80
199	1016	14.40	121.00	5.76	159.51	X80	638.00	739.00	20.80
200	1016	14.40	121.00	7.70	159.51	X80	638.00	739.00	20.00
201	1016	14.40	121.00	8.64	159.51	X80	638.00	739.00	18.20
202	1016	14.40	121.00	10.08	159.51	X80	638.00	739.00	16.40
203	1016	14.40	121.00	11.52	159.51	X80	638.00	739.00	13.60
204	1422	14.40	143.00	2.88	223.25	X80	638.00	739.00	17.00
205	1422	14.40	143.00	4.32	223.25	X80	638.00	739.00	15.80
206	1422	14.40	143.00	5.76	223.25	X80	638.00	739.00	14.00
207	1422	14.40	143.00	7.70	223.25	X80	638.00	739.00	13.00
208	1422	14.40	143.00	8.64	223.25	X80	638.00	739.00	11.00
209	1422	14.40	143.00	10.08	223.25	X80	638.00	739.00	8.80
210	1422	14.40	143.00	11.52	223.25	X80	638.00	739.00	8.00
211	610	8.10	39.60	2.45	31.90	X80	601.00	684.00	19.34
212	610	8.10	39.60	2.45	31.90	X80	601.00	684.00	20.45
213	610	8.10	39.60	2.45	31.90	X80	601.00	684.00	20.76
214	610	8.10	39.60	2.45	31.90	X80	601.00	684.00	21.56
215	610	8.10	39.60	2.45	31.90	X80	601.00	684.00	22.06
216	610	8.10	39.60	2.45	31.90	X80	601.00	684.00	22.14
217	610	8.10	39.60	2.45	31.90	X80	601.00	684.00	22.18
218	610	8.10	39.60	2.45	31.90	X80	601.00	684.00	22.20
219	610	8.10	39.60	2.45	31.90	X80	601.00	684.00	22.21
220	610	8.10	39.60	4.90	31.90	X80	601.00	684.00	16.75
221	610	8.10	39.60	4.90	31.90	X80	601.00	684.00	18.57
222	610	8.10	39.60	4.90	31.90	X80	601.00	684.00	19.72
223	610	8.10	39.60	4.90	31.90	X80	601.00	684.00	20.85
224	610	8.10	39.60	4.90	31.90	X80	601.00	684.00	21.52
225	610	8.10	39.60	4.90	31.90	X80	601.00	684.00	21.31
226	610	8.10	39.60	4.90	31.90	X80	601.00	684.00	21.61
227	610	8.10	39.60	4.90	31.90	X80	601.00	684.00	21.65
228	610	8.10	39.60	4.90	31.90	X80	601.00	684.00	21.66
229	458.8	8.10	39.60	7.35	31.90	X80	601.00	684.00	13.79
230	458.8	8.10	39.60	7.35	31.90	X80	601.00	684.00	15.15
231	458.8	8.10	39.60	7.35	31.90	X80	601.00	684.00	16.47
232	458.8	8.10	39.60	7.35	31.90	X80	601.00	684.00	18.21
233	458.8	8.10	39.60	7.35	31.90	X80	601.00	684.00	18.78
234	458.8	8.10	39.60	7.35	31.90	X80	601.00	684.00	19.29
235	458.8	8.10	39.60	7.35	31.90	X80	601.00	684.00	19.81
236	458.8	8.10	39.60	7.35	31.90	X80	601.00	684.00	19.84
237	458.8	8.10	39.60	7.35	31.90	X80	601.00	684.00	19.87
238	458.8	8.10	39.60	5.39	32.10	X80	638.00	739.00	20.90
239	458.8	8.10	39.60	5.39	32.10	X80	638.00	739.00	21.20
240	458.8	8.10	39.60	5.39	32.10	X80	638.00	739.00	21.10
241	458.8	8.10	39.60	5.39	32.10	X80	638.00	739.00	17.10
242	458.8	8.10	39.60	5.39	32.10	X80	638.00	739.00	21.50
243	458.8	8.10	39.60	5.39	32.10	X80	638.00	739.00	15.20
244	458.8	8.10	39.60	5.39	32.10	X80	638.00	739.00	22.00
245	458.8	8.10	39.60	5.39	32.10	X80	638.00	739.00	22.00
246	458.8	8.10	39.60	5.39	32.10	X80	638.00	739.00	23.50
247	458.8	8.10	39.60	5.39	32.10	X80	638.00	739.00	24.00
248	458.8	8.10	39.60	5.39	32.10	X80	638.00	739.00	20.00
249	458.8	8.10	39.60	5.39	32.10	X80	638.00	739.00	24.40
250	458.8	8.10	39.60	5.39	32.10	X80	638.00	739.00	16.00
251	458.8	8.10	39.60	5.39	32.10	X80	638.00	739.00	20.00
252	458.8	8.10	39.60	5.39	32.10	X80	638.00	739.00	20.50
253	458.8	8.10	39.60	5.39	32.10	X80	638.00	739.00	20.80
254	458.8	8.10	39.60	5.39	32.10	X80	638.00	739.00	21.00
255	458.8	8.10	39.60	5.39	32.10	X80	638.00	739.00	21.20
256	458.8	8.10	39.60	5.39	32.10	X80	638.00	739.00	21.40
257	458.8	8.10	39.60	5.39	32.10	X80	638.00	739.00	21.50
258	458.8	8.10	39.60	5.39	32.10	X80	638.00	739.00	21.70
259	458.8	8.10	39.60	5.39	32.10	X80	638.00	739.00	18.80
260	458.8	8.10	39.60	5.39	32.10	X80	638.00	739.00	23.20
261	458.8	8.10	39.60	5.39	32.10	X80	638.00	739.00	23.50
262	458.8	8.10	39.60	5.39	32.10	X80	638.00	739.00	23.70
263	458.8	8.10	39.60	5.39	32.10	X80	638.00	739.00	23.90
264	458.8	8.10	39.60	5.39	32.10	X80	638.00	739.00	24.10
265	458.8	8.10	39.60	5.39	32.10	X80	638.00	739.00	24.30
266	458.8	8.10	39.60	5.39	32.10	X80	638.00	739.00	24.50
267	458.8	8.10	39.60	5.39	32.10	X80	638.00	739.00	24.50
268	458.8	8.10	39.60	2.45	31.90	X80	601.00	684.00	23.84
269	458.8	8.10	39.60	2.45	31.90	X80	601.00	684.00	22.73
270	458.8	8.10	39.60	2.45	31.90	X80	601.00	684.00	24.61

## References

[1] Rubaiee S. (2023). High sour natural gas dehydration treatment through low temperature technique: Process simulation, modeling and optimization. Chemosphere.

[2] Vosikovsky O., Macecek M., Ross D.J. (1983). Allowable defect sizes in a sour crude oil pipeline for corrosion fatigue conditions. Int. J. Press. Vessel. Pip..

[3] Li X., Ma X., Zhang J., Akiyama E., Wang Y., Song X. (2020). Review of Hydrogen Embrittlement in Metals: Hydrogen Diffusion, Hydrogen Characterization, Hydrogen Embrittlement Mechanism and Prevention. Acta Metall. Sin. (Engl. Lett.).

[4] Cai L., Bai G., Gao X., Li Y., Hou Y. (2022). Experimental investigation on the hydrogen embrittlement characteristics and mechanism of natural gas-hydrogen transportation pipeline steels. IOP Publ. Ltd..

[5] Negi A., Barsoum I., Alfentanil A. (2023). Predicting sulfide stress cracking in a sour environment: A phase-field finite element study. Theor. Appl. Fract. Mech..

[6] Shimamura J., Morikawa T., Yamasaki S., Tanaka M. (2022). Sulfide stress cracking (ssc) of low alloy linepipe steels in low h_2s content sour environment. ISIJ Int..

[7] Dong S., Zhang L., Zhang H. (2021). Crack propagation rate of hydrogen-induced cracking in high sulfur-containing pipelines. Eng. Fail. Anal..

[8] Khaksar L., Shirokoff J. (2017). Effect of elemental sulfur and sulfide on the corrosion behavior of cr-mo low alloy steel for tubing and tubular components in oil and gas industry. Materials.

[9] Cao J. (2024). Effect of hydrogen embrittlement on the safety assessment of low-strength hydrogen transmission pipeline. Eng. Fail. Anal..

[10] Xu Y. (2022). Adaptability Analysis of Residual Strength Evaluation Methods for Corroded Pipelines. Chem. Saf. Environ. Prot..

[11] Li H., Huang K., Zeng Q., Sun C. (2022). Residual strength assessment and residual life prediction of corroded pipelines: A decade review. Energies.

[12] Zuo L., Zeng C., Hu X., Du S., Zhao Y., Fei F. (2022). Evaluation of corrosion residual life prediction methods for metal pipelines. Materials.

[13] Yuan Y., Deng K., Zhang J., Zeng W., Kong X., Lin Y. (2021). Finite element study on residual internal pressure strength of corroded oil pipes and prediction method for remaining life. Anti-Corros. Methods Mater..

[14] Li Q., Li Z. (2024). Research on failure pressure prediction of water supply pipe based on ga-bp neural network. Water.

[15] Zhang L., Luo Y., Shen Z., Ye D., Li Z. (2024). Optimization design of the elbow inlet channel of a pipeline pump based on the scso-bp neural network. Water.

[16] Xin J., Chen J., Li C., Lu R.-K., Li X., Wang C., Zhu H., He R. (2021). Deformation characterization of oil and gas pipeline by acm technique based on ssa-bp neural network model. Measurement.

[17] Jia Z., Ren L., Li H., Sun W. (2018). Pipeline leak localization based on fbg hoop strain sensors combined with bp neural network. Appl. Ences.

[18] Ji H., Zhang X., Wang T., Yang K., Jiang J., Xing Z. (2025). Oil spill area prediction model of submarine pipeline based on bp neural network and convolutional neural network. Process. Saf. Environ. Prot..

[19] Li X., Jing H., Liu X., Chen G., Han L. (2023). The prediction analysis of failure pressure of pipelines with axial double corrosion defects in cold regions based on the bp neural network. Int. J. Press. Vessel. Pip..

[20] Li N., Jia B., Chen J., Sheng Y., Deng S. (2023). Phenomenological 2d and 3d models of ductile fracture for girth weld of x80 pipeline. Buildings.

[21] Fan W., Gao J., Liu C. (2018). Effect of Localized Corrosion Depth on the Performance of L360 Pipelines under High Acidity Conditions. Oil Gas Storage Transp..

[22] Westergaard H.M. (1952). Theory of Elasticity and Plasticity.

[23] Johnson R., Cook W.K. A Constitutive Model and Data for Metals Subjected to Large strains High Strain Rates and High Temperatures. Proceedings of the 7th International Symposium on Ballistics.

[24] Sun M., Zhao H., Li X., Liu J., Xu Z. (2021). New evaluation method of failure pressure of steel pipeline with irregular-shaped defect. Appl. Ocean. Res..

[25] Mensah A., Sriramula S. (2023). Estimation of burst pressure of pipelines with interacting corrosion clusters based on machine learning models. J. Loss Prev. Process Ind..

[26] Benjamin A., Freire J., Vieira R. (2007). Part 6: Analysis of pipeline containing interacting corrosion defects. Exp. Tech..

[27] Holland J.H. (1975). Adaptation in Natural and Artificial Systems.

[28] Aivaliotis-Apostolopoulos P., Loukidis D., Mirjalili S. (2022). Swarming genetic algorithm: A nested fully coupled hybrid of genetic algorithm and particle swarm optimization. PLoS ONE.

[29] Qing S., Dai Q., Guo H. (2018). Prediction of Ultimate Pressure of Corroded Defect Pipelines Based on BP Neural Network. Corros. Prot..

[30] Kim W.-S., Kim Y.-P., Kho Y.-T., Choi J.-B. Full scale burst test and finite element analysis on corroded gas pipeline. Proceedings of the 2002 4th International Pipeline Conference.

[31] Kim Y.-P., Kim W.-S., Lee Y.-K., Oh K.-H. The evaluation of failure pressure for corrosion defects within girth or seam weld in transmission pipelines. Proceedings of the 2004 International Pipeline Conference.

[32] Freire J., Vieira R., Castro J., Benjamin A. (2010). Part 3: Burst tests of pipeline with extensive longitudinal metal loss. Exp. Tech..

[33] Benjamin A.C., Freire J.L., Vieira R.D., Diniz J.L., de Andrade E.Q. Burst tests on pipeline containing interacting corrosion defects. Proceedings of the Asme International Conference on Offshore Mechanics & Arctic Engineering.

[34] Chen J.M., Meng H.M., Li Y.Y. Pipeline prescription analysis after corrosion and explosive test. Proceedings of the China International Corrosion Control Conference’99.

[35] Shuai Y., Shuai J., Liu C.Y. (2017). Research on the reliability methods of corroded pipeline. Pet. Sci. Bull..

[36] Xiang Y., Zhu L., Jia B., Zhao L., Li N., Gu Y., Ren P. (2025). Sensitivity analysis and failure prediction of x80 pipeline under transverse landslide. J. Constr. Steel Res..

[37] Wei W., Cong R., Li Y., Abraham A.D., Yang C., Chen Z. (2022). Prediction of tool wear based on GA-BP neural network. Proc. Inst. Mech. Eng. Part B J. Eng. Manuf..

[38] Mcculloch W.S., Pitts W. (1943). A logical calculus of the ideas immanent in nervous activity. Bull. Math. Biophys..

[39] Rahmawati S.D., Santoso R.K., Tanjungsari F. (2021). Integrated CO_2_-H_2_S corrosion-erosion modeling in gas production tubing and pipeline by considering passive layer activity. J. Pet. Explor. Prod. Technol..

[40] Guzenkova A.S., Artamonova I.V., Guzenkov S.A., Ivanov S.S. (2021). Steel Corrosion in Hydrogen Sulfide Containing Oil Field Model Media. Metallurgist.

[41] Kanchanomai C., Phiphobmongkol V., Muanjan P. (2008). Fatigue failure of an orthopedic implant—A locking compression plate. Eng. Fail. Anal..

[42] Okonkwo P.C., Shakoor R.A., Benamor A., Amer Mohamed A.M., Al-Marri M.J. (2017). Corrosion behavior of api x100 steel material in a hydrogen sulfide environment. Metals.

[43] Tian Y., Li S., Xiao J. (2020). Research on Internal Corrosion Prediction Model for Sulfur-containing Natural Gas Gathering and Transportation Pipelines. Oilfield Surf. Eng..

[44] Tian Y., Li S., Xiao J. (2021). Application of Corrosion Prediction Software for Sulfur-containing Gas Field Gathering and Transportation Pipelines. Pet. Nat. Gas Chem. Ind..

[45] Yang J., Ma G. (2006). Prediction Methods for Corrosion of Sulfur-containing Pipelines. Oil Gas Storage Transp..

[46] Liu D., Liang F., Gong J., Zhao J. (2012). Prediction of Corrosion Rate of L360 Steel in High-sulfur Gas Fields Based on Neural Network. Petrochem. Corros. Prot..

[47] Wang Z., Lan X. (2011). Corrosion Behavior of L360 Steel in High-pressure H_2_S/CO_2_ Gas-liquid Two-phase Environment. Corros. Prot..

[48] (2010). Pipeline External Corrosion Direct Assessment Methodology.

